# The Educated Citizen and Global Public-Health Issues: One Model for Integration into the Undergraduate Curriculum

**DOI:** 10.3389/fpubh.2016.00035

**Published:** 2016-03-01

**Authors:** Rosemary M. Caron

**Affiliations:** ^1^Department of Health Management and Policy, College of Health and Human Services, University of New Hampshire, Durham, NH, USA

**Keywords:** undergraduate public health, global public health, refugees, liberal education, public health

## Abstract

The Educated Citizen Initiative proposes that an understanding of public-health issues is a core component of an educated citizenry and is essential to develop one’s societal responsibility. This initiative supports the Institute of Medicine’s recommendation that “all undergraduates should have access to education in public health.” Furthermore, the Liberal Education and America’s Promise (LEAP) framework developed by the Association of American Colleges and Universities supports the “integration of public-health education into general and liberal education with an aim to produce an educated citizenry.” The LEAP framework is implemented by teaching about the role of social determinants in a population’s health status; the significance of personal and social responsibility; and providing skills for inquiry, critical thinking, problem solving, and evaluation. This article describes one university’s experience in generating an educated citizenry cognizant of comprehensive public-health conflicts, thus contributing to both a local and global perspective on learning.

## Public Health and Liberal Education

Winslow, often referred to as the Father of Public Health, defined public health as:

… the science and art of preventing disease, prolonging life and promoting physical health and efficacy through organized community efforts for the sanitation of the environment, the control of community infections, the education of the individual in principles of personal hygiene, the organization of medical and nursing services for the early diagnosis and preventive treatment of disease, and the development of the social machinery which will ensure every individual in the community a standard of living adequate for the maintenance of health … to enable every citizen to realize his or her birthright and longevity (1).

The Institute of Medicine (IOM) of the United States’ National Academy of Sciences defines public health as: “fulfilling society’s interest in assuring conditions in which people can be healthy” (2). Thus, public health’s main mission is to promote health, prevent disease, and protect human populations. In order to achieve this mission, public health operates at various levels of government, private, and non-profit sectors.

In 2003, the IOM stated that a well-educated public-health workforce was essential to keep the public healthy. As a result, the IOM recommended that “all undergraduates should have access to education in public health” (3). The IOM further challenged academia that undergraduate public health should be viewed not solely as a professional credential but as a part of the educator’s responsibility to help develop educated citizens (3).

Albertine et al. stated that the significance of developing a liberal arts curriculum is to educate students, the future citizenry, about key public-health principles:

We need citizens who can help as individuals to change social behavior and who are aware of the need for systemic health care, good nutrition, decent housing, and sustainable urban centers. We need to rely on leaders who are able to consider benefits and harms to groups, minority as well as majority, and to engage in systems thinking, understanding how multiple factors interact. These are abilities essential to citizenship for the health of the world (4).

The Educated Citizen and Public Health Initiative, which was developed by the Association of American Colleges and Universities (AAC&U), the Association for Prevention Teaching and Research, and the Council on Colleges of Arts and Sciences, is the response to the IOM’s call that “all undergraduates should have access to education in public health” (3). The Educated Citizen Initiative supports that an understanding of public-health issues is a core component of a public who has been educated, and it is necessary to develop one’s social duty (5).

Liberal education can also contribute to this call-to-action to educate citizens who can think critically and pull from different disciplines to develop practical recommendations on complex public-health issues. Liberal education is a “… philosophy of education that empowers individuals with core knowledge and transferable skills and cultivates social responsibility and a strong sense of ethics and values” (6). AAC&U’s Liberal Education and America’s Promise (LEAP) report proposes a joint approach that utilizes the strengths of a liberal education and public-health education; one, “… that advocates for integrative, interdisciplinary, and applied knowledge and practice, for community outreach and civic responsibility across all undergraduate programs, for global awareness and responsibility, and for open pathways among the arts and sciences and professional schools and between the campus and the wider world. The campaign underscores the importance of learning for a free society and development of human talent” (7).

Albertine et al. stated that “We need citizens who possess an ability to think about the big picture, beyond the individual or the constituency” (4). Thus, the integration of public health into liberal education will help to generate an educated citizen who possesses “a wide breadth of knowledge, adaptable skills, principled values and a sense of societal responsibility” (8). The purpose of this article is to demonstrate how one course, Global Public Health Issues, is contributing toward the education of a citizenry that thinks critically about their fellow citizen from a local and global perspective.

## Global Public Health

The University of New Hampshire (hereinafter referred to as the “University”) is a public university founded in 1866 and is located in Durham, NH, USA. The University is educating approximately 13,000 undergraduate students and 2,500 graduate students. More than 200 degree programs are offered on the University’s three campuses (9).

The course titled, Global Public Health Issues, is offered by the Department of Health Management and Policy that offers the following courses/program: a Bachelor of Science degree with undergraduate courses specific to health administration and public health, a minor in Public Health (but not a major in Public Health), and a graduate Master’s in Public Health Program. The Global Public Health Issues course is taught by a tenured, full professor, and is taught once per year (fall semester). Originally, this course was open to freshmen and allowed for a small enrollment of 20 students to assist with the acclimation of new students to college courses where they could “find their voice” in a smaller class and not be intimidated by upperclassmen. More recently, this course has been open to Honors students participating in the Honors Program Symposium (see below for discussion) at the University. Honors students need to meet certain criteria, including having a 3.40 minimum grade point average, to be invited to participate in such a program. Honors courses are capped at 20 students, so the class size is small, relatively speaking, for a class size at this public academic institution. The course is taught twice a week in 80-min sessions for the duration of the semester (15 weeks), thus allowing for approximately 28 in-person class sessions due to federal and state holidays (e.g., Labor Day, Veteran’s Day) that are observed.

Using the perspective of public health, the course covers factors associated with the development of health problems and efforts to prevent disease in impoverished areas. Students also explore the role of social communication, politics, religion, economics, education, and culture in contributing to global public-health issues and integrate these factors and values in developing solutions to the widespread public-health issues impacting communities worldwide. Students learn about the magnitude of public-health issues in the developing world (e.g., communicable and non-communicable disease, women and child health, nutrition, and unintentional injuries), how health is assessed, and how health systems work together effectively to improve global health. Box 1 highlights the anticipated knowledge, skills, and values that the student will acquire by the end of the course, including how these attributes will be achieved.

Box 1 **Course objectives for global public health issues.^1^**

A. Knowledge

At the conclusion of the course, students should be able to describe and generally assess the following:

1. Key public-health concepts (e.g., demographics, determinants of health, epidemiologic transition of disease, measures of health status, etc.).
2. The burden of disease in developing countries and its impact on a community’s health.
3. Critical issues in the organization and delivery of health services.
4. Impact of globalization on the spread of disease.
5. Role of social and cultural factors in affecting a society’s vulnerability to morbidity and mortality.
6. Critical issues in the organization and delivery of public-health and health-care services to control and prevent morbidity and mortality.

B. Skills

After taking this course, students will be able to:

1. Describe methods used to assess population health.
2. Describe organized efforts to address the health of developing countries.
3. Identify links among health, social, and economic factors that affect population health.

C. Values

At the conclusion of this course, students will:

1. Appreciate key factors in global health and how they interact to address critical public-health issues.
2. Appreciate the interconnectedness of a community’s health with the health of the social, political, economic, and physical environment.

The schematic below illustrates the inter-relationship among the knowledge, skills, and values taught in the course and the student assessment methods (which are highlighted in the narrative and Box 2).

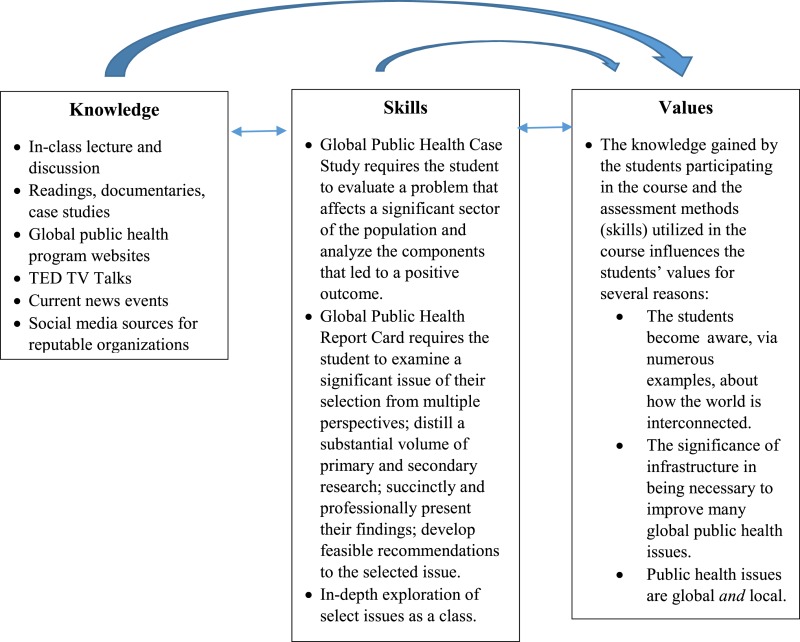

^1^These course objectives are based on Riegelman RK, Albertine S, Persily NA, Kaelin MW, Cashman S. *Curriculum Guide for Undergraduate Public Health Education*. Available at http://c.ymcdn.com/sites/www.aptrweb.org/resource/resmgr/undergraduateph/curriculum_guide_version3.pdf

This course is taught in a seminar format utilizing a mixture of teaching methods including lecture, class discussion, group work, and the incorporation of literature and the arts to illustrate central themes and course principles. The course assessments include written work in essay-format exams; analysis and synthesis of course material and peer-reviewed literature to propose feasible solutions to complex global public-health issues *via* a case-study method; and a comprehensive scenario-based assignment that requires the student to orally present (as part of a team) a relevant global public-health issue and to develop practical recommendations at the government, community, and individual levels.

## Honors Program Symposium

In the 2015–2016 academic year, the Honors Program at the University developed interdisciplinary “supercourses” that were composed of four courses and each met the University’s Discovery Program designation (10). The Discovery Program is “the core curriculum that provides students a solid foundation for inquisitive problem solving, scientific reasoning, an appreciation of the arts and humanities, research skills, and communication. It is based on a breadth of courses in a variety of disciplines that the faculty collectively believe are essential to a liberal education. Courses within the eight disciplinary categories expose students to topics in the arts, humanities, philosophy, social sciences, physical and biological sciences, and technology to prepare them with transferable skills for a lifetime of learning and creative endeavors as globally focused, socially responsible citizens in the world in which they live” (11).

The interdisciplinary courses that comprised the Honors Program Symposium were each taught by one faculty member and they convened five times over the duration of the semester for plenary sessions in which “distinct disciplinary approaches” to examine a particular issue or problem was modeled (10). Examples included a faculty panel discussion regarding avian flu; participation in an international health-focused University-wide seminar series; viewing a film on tuberculosis in Swaziland followed by an in-class, interdisciplinary group discussion; and lastly, student-created videos on any topic related to the Honors Program Symposium. The goal was to have all students participate in a common Honors experience with an interdisciplinary focus.

The Global Public Health Issues course is one of the courses in the first Honors Program Symposium offered by the University which is focused on the following theme: reinventing healthy communities nationally and globally: medical, legal, and cultural perspectives. The rationale for this theme is as follows. “Communities are comprised of a social fabric that connects diverse populations and their access and utilization of health care, and their attainment and maintenance of health. This Honors Symposium will engage Honors students in the study of community and the health of the constituents who reside in these communities *via* multidisciplinary lenses that include the following: (1) comparing public-health issues in developed versus developing countries with varied health systems and analyzing the root causes that account for these differences; (2) examining how individuals have and do relate to, and engage in, a changing health-care system that is influenced by not only medicine but also by science, law, and society; (3) considering how techno-scientific developments, transnational flows, environmental transformations, and historical inequities intersect to shape how individuals know and experience their bodies cross-culturally; and (4) developing a value-based understanding of personal accountability and social advocacy in the community context. Together, these courses will enable today’s student to be an active participant in their community and to provide them with the knowledge and skills to ‘think globally and acting locally’ as we pursue health for all” (12).

## Global Public Health Student Assessments

In general, there were three student assessments implemented throughout the semester.

### Global Public Health Case Study

Students select a case study from peer-reviewed sources [e.g., *American Journal of Public Health* articles and/or *Ruth Levine’s Case Studies in Global Health: Millions Saved* published (2007) by Jones and Bartlett Publishers]. Each evidence-based case study describes how major public-health efforts can and have changed the health of populations around the globe. Once a case study has been selected, the student is provided a series of questions for completion. The questions are thought-provoking and require the students to utilize information from the case itself, as well as conduct research about the public-health issue to answer the questions.

### Global Public Health Report Card

A major assignment for the course is a Global Public Health Report Card where the student is presented with a scenario and must consider their selected public-health issue from a multidisciplinary perspective. This assignment is highlighted in Box 2.

Box 2 **Global public health assessments.**

Global Public Health Report Card

You are working for the World Health Organization and you have been given 30 min to meet the Director-General to provide a brief overview of your progress in improving global health.

To make the most of the limited time with the Director-General, you have decided to develop a “snapshot” of the global public-health issue for your selected geographic region in the form of a Global Public Health Report Card.

This Global Public Health Report Card should be concise (no more than 10 pages), well-developed, and utilize graphics (e.g., tables, charts, maps, photos).

At minimum, the following topics should be presented in the Global Public Health Report Card:

1. An executive summary in one paragraph that summarizes all the points you want to make.
2. Describe the major public-health issue of concern.
3. Description of the population most affected by this public-health issue (including demographics and the health status of the community).
4. Description of the region’s social, physical, economic, political, and cultural environments.
5. Specific to this public-health problem, how does the community’s health compare to specific standards, e.g., Healthy People 2020, WHO, CDC.
6. The major stakeholders in this specific public-health problem.
7. What is the community/government doing to address the major public-health problem? Are they doing enough, in your opinion? Explain your position.
8. Recommendations for the community and the individual (describe at least two specific recommendations for each).
9. Cost and consequences of this global public-health problem to the community/region/country.
10. Your recommendations to the Director-General on how the health of this specific population can be improved; propose at least two specific interventions.

Global Public Health Essay Exams

The following are representative short essay exam questions requiring the student to recall course materials (e.g., readings, text chapters, lecture notes, class discussions, and documentaries) to specifically and concisely answer each question:

1. Describe how public health differs from medical care.
2. Compare, by providing at least three reasons, why polio eradication efforts are more challenging than the approach utilized in the smallpox eradication program.
3. As countries develop economically, briefly explain the most important changes that occur in their burden of disease and the reasons for these changes.
4. Describe the health-care and public-health systems of two different countries.
5. Discuss three human rights issues pertaining to people with a stigmatized disease.
6. Identify and describe the four health behavior change models discussed in class.
7. Using one of the health behavior change models discussed in class, briefly describe how you would encourage the adoption of a healthy behavior in a large sector of the population.
8. Which environmental health issue discussed in class is the most important and why?
9. Explain how women are not the problem but can be the solution to a developing country’s prosperity. Include at least two examples from our class discussions/readings.
10. Identify the Millennium Development Goals and briefly describe the significance of each.

### Global Public Health Essay Exams

Two in-class exams and a cumulative final are implemented in the course. These exams are short essay in design and require the student to recall course materials (e.g., readings, text chapters, lecture notes, class discussions, and documentaries) to specifically and concisely answer each question. Box 2 highlights representative exam questions.

## Global Public Health Issues

In addition to the assessment methods referenced above, three specific global public-health issues were examined in-depth in this course: childhood lead poisoning, refugee resettlement, and complex humanitarian emergencies. These issues not only emphasize the theme of the Honors Program Symposium but also highlight the relationship between public health and liberal education.

- The first conflict, childhood lead poisoning is presented as a wicked problem that is multifactorial and possesses no obvious resolution due to the numerous stakeholders and their varied interests (13). This case stems from the faculty member’s involvement in a pediatric fatality of a 2-year-old Sudanese refugee child in a northern New England community, hence, how a local public-health issue can be a problem for refugees. At the time, the Centers for Disease Control and Prevention’s action level for childhood lead poisoning was 10 μg/dl. This refugee child presented with an elevated blood lead level of 391 μg/dl (14). In this case, childhood lead poisoning “illustrates how understanding a community’s ecology can build community capacity to affect local environmental management by (1) forming an academic–community partnership and (2) developing a place-specific strategy grounded in the cultural–experiential model of risk” (13). The students develop feasible, primary, secondary, and tertiary prevention methods for this complex public-health issue in the African refugee community, a disproportionately affected population.
- The second conflict addressed in the course is the refugee resettlement model in general, and specifically in New Hampshire. The lens used to examine this issue is that “communities are important health determinants for resettled refugees. The risk for lead poisoning among African refugee children who resettle in the United States remains elevated, despite the gradual decrease in childhood lead poisoning in this country. I argue that the refugee resettlement process is a restricted system with a limited infrastructure that inadvertently contributes to the disproportionate burden of lead poisoning cases experienced by resettled African refugee children” (14). Childhood lead poisoning in a resettled African refugee population is presented as a case study of environmental inequality. The students research the process in other states and countries and propose practical recommendations for public-health practitioners and stakeholders to reduce and ultimately eliminate this unintended environmental inequality.
- The third conflict examined in the course is the migration of immigrants from Syria to other countries. The students examine the management of this complex humanitarian emergency *via* scholarly articles and news media (including social media sources). The students then develop and submit a Letter to the Editor of a news source they prefer, stating their rationale for their opinion and proposed resolution of this unprecedented tragedy. Class time is set aside to discuss and debate the varied perspectives on this developing issue.

## Conclusion

Educators need to be creative in responding to the IOM’s call to action that “all undergraduates should have access to education in public health” (3). This article highlights how a course focused on global public-health issues can utilize the features of a liberal education to help develop an educated citizenry by having students examine public-health issues that may be local but are affecting a global population and how global issues have the potential, and in some instances are affecting one’s local community. Furthermore, academic institutions should consider how to use existing courses to develop programs, such as the Honors Symposium described herein, to promote an interdisciplinary approach to solving complex public-health issues, as well as provide public-health education.

Students enrolled in this course evaluated the course and its role in the Honors Symposium favorably. The students were prompted to provide comments related to course content, grading, or structure. A few select student comments include the following:

- “I took this class to fulfill a discovery requirement and it has had such a tremendous and positive influence on me. I had no experience with public health and now having taken this course, I look at the world and its various health issues very differently and I realize how important it is to have an awareness of public health, whereas before this class I did not.”
- “There was never a class that I didn’t leave feeling like my eyes had been opened to something I had never considered and that I had learned something that made me want to change the world.”
- (This course made me care) “deeply about [my] community, locally and globally. If it wasn’t for already having a direction for a career, I would seriously consider going into public health … but I know for the rest of my life it will be a deep interest of mine and I will absolutely do what I can to better the lives of people who do not have the good health that I am able to enjoy.”

Hill et al. surveyed 50 liberal arts colleges with respect to their offering a course, program, track, or concentration in global public health. The authors reported that all colleges surveyed offered one course with a global public-health theme (15). The authors stated that the “Values of a liberal arts education are found in the study of global and public health: social responsibility, critical thinking, ethical reasoning, and knowledge of the wider world … Capturing interest in global public health will enhance the curriculum and student experience” (15). I support this view, based on my own experience with teaching this introductory course in Global Public Health Issues. Future plans involve offering this course at my academic institution more than once per year to not only Honors students but also all classes of students to allow for an introduction to the significance of public-health issues globally and locally *via* the lens of liberal education. A consideration to this proposal is that the assessment methods, as currently developed, are labor intensive on the part of the student completing the work and the faculty member grading the work. Thus, the assessment methods would require revision should the course be scaled to allow for larger class sizes.

The future implications of this effort are that the professional knowledge and skills obtained through such a model will result in not only an educated population but also one who can make informed decisions and participate on a personal level in activities that promote health not only for themselves and their families but also their community, state, nation, and the world in which they live.

## Author Contributions

The author is the sole, corresponding author of the work described in this manuscript. She has designed and implemented the work described. The perspective provided in the manuscript is her own.

## Conflict of Interest Statement

The author declares that the research was conducted in the absence of any commercial or financial relationships that could be construed as a potential conflict of interest.
